# Associations between time spent with digital media and body image among European adolescents

**DOI:** 10.1186/s12887-026-06551-w

**Published:** 2026-01-31

**Authors:** Gowsiga Loganathan, Christoph Buck, Garrath Williams, Toomas Veidebaum, Michael Tornaritis, Dénes Molnár, María L Miguel-Berges, Lauren Lissner, Annarita Formisano, Stefaan De Henauw, Joanna Baran, Antje Hebestreit, Elida Sina, I.Family consortium

**Affiliations:** 1 Leibniz Institute for Prevention Research and Epidemiology – BIPS, Bremen, Germany; 2 Department of Politics, Philosophy & Religion, Lancaster University, Lancaster, UK; 3 Department of Chronic Diseases, National Institute for Health Development, Tallinn, Estonia; 4 Research and Education Institute of Child Health, Strovolos, Cyprus; 5 Department of Paediatrics, Medical School, University of Pécs, Pécs, Hungary; 6 Growth, Exercise, NUtrition and Development (GENUD-B34_23R) Research Group, Instituto Agroalimentario de Aragón (IA2), Universidad de Zaragoza and Instituto de Investigación Sanitaria de Aragón (IIS Aragón), Zaragoza, Spain; 7 Centro de Investigación Biomédica en Red de Fisiopatología de la Obesidad y Nutrición (CIBERObn), Instituto de Salud Carlos III, Madrid, Spain; 8 School of Public Health and Community Medicine, Institute of Medicine, Sahlgrenska Academy, University of Gothenburg, Gothenburg, Sweden; 9 Institute of Food Sciences, National Research Council, Avellino, Italy; 10 Department of Public Health and Primary Care, Faculty of Medicine and Health Sciences, Ghent University, Ghent, Belgium; 11 Institute of Physiotherapy, Department of Health Sciences and Psychology, Medical College, University of Rzeszów, Rzeszów, Poland; 12 Institute for Evidence in Medicine, Medical Center and Faculty of Medicine, University of Freiburg, Freiburg, Germany

**Keywords:** Body image, Digital media, Adolescents, Latent class analyses

## Abstract

**Background:**

External factors, including digital media (DM), promote body ideals that can shape adolescents’ body image, but studies across European countries are scarce. Therefore, the aim of the study was to examine the relationship between daily DM duration and body image dissatisfaction (BID) in adolescents from nine European countries.

**Methods:**

Participants from the I.Family study self-reported daily DM duration and BID in 2013/2014 (*n* = 3,608; 51% female; mean age 13.6 years (standard deviation: 1.1). DM duration was measured in hours/day, including television viewing (TV), computer/game console (PC), smartphone, and internet use. Linear regression models were used to examine associations of self-reported DM duration with BID and unstandardised regression coefficients were reported. Daily time spent with these technologies was categorised into < 1, 1–2, and ≥ 2 h, and underlying patterns of DM use were identified using latent class analyses. Furthermore, the interaction term between family environment and DM was included in the latent class analyses.

**Results:**

Increasing daily DM duration, particularly for smartphone (adjusted β = 0.44, 95%CI: 0.31, 0.57) and internet (adjusted β = 0.40, 95%CI: 0.29, 0.50), was associated with higher BID in all adolescents. Associations were more pronounced in underweight, normal weight, and female participants. Adolescents with high internet and smartphone duration in combination with medium/low TV/PC duration showed higher positive associations with BID score compared to those with low duration of all DM types (adjusted β = 1.24, 95%CI: 0.73, 1.74). A positive family environment attenuated the association in adolescents with high internet/smartphone and medium/low TV/PC duration.

**Conclusion:**

The results highlight a positive association between longer daily DM duration and BID in adolescents, especially for internet-enabled media. A positive family environment seems to play a role in this association and should be further investigated in future research. Additionally, understanding the potential mechanisms explaining these associations can inform future interventions promoting healthy body image in adolescents.

**Supplementary Information:**

The online version contains supplementary material available at 10.1186/s12887-026-06551-w.

## Background

 Body image is a complex construct with multiple dimensions, including a behavioural dimension involving body-related actions, a perceptual dimension involving the evaluation of body attributes, and cognitive-emotional aspects involving thoughts directed towards one’s body [1, 2]. Body image dissatisfaction (BID) is the perception of the discrepancy between one’s body perception and the idealised body, i.e., the desired body [2, 3].

Adolescence, a sensitive period of physical, psychological, and social transitions, is particularly vulnerable to body image issues [4, 5]. During puberty, especially in females, body changes are sometimes associated with unintentional weight gain, contrary to the societal ideals of thinness [6]. Similarly, male adolescents may experience body dissatisfaction and often seek a lean and muscular physique [5]. In addition, adolescents with overweight/obesity may also experience increased BID, due to greater discrepancies between their bodies and ‘ideal’ body conceptions [5, 7].

The development of body ideals is influenced by external factors, including family, friends, and mass media, as proposed in the tripartite influence model [6, 8]. However, with the increasing use of digital media (DM) over age in particular [9], idealised body images are now ubiquitously promoted to adolescents at any place and time, increasing pressure on unrealistic beauty standards [10, 11].

Previous research has shown that appearance-based content on DM can contribute to increased BID, particularly among female adults and adolescents [10]. Importantly, both male and female adolescents are exposed to specific body ideals through different DM, like music posts, videos or games [10]. Nonetheless, research on DM use and body image in male adolescents remains limited and inconclusive [11, 12]. Most of the previous studies conducted have been female-centred, as body image has traditionally been considered a female-oriented topic. Furthermore, the studies conducted in male adolescents were small in sample size or did not focus on DM types mostly used by male adolescents, such as gaming [13, 14].

Additionally, most of the studies investigating how DM influences body image are conducted in the USA and Australia, with limited data available from and across European countries. Also, most studies have not considered multiple DM types, although usage patterns have changed such that adolescents use more than just one DM. Existing evidence has also not sufficiently explored differences by weight status in the association between DM use and body image in adolescents.

Moreover, when investigating the role of DM on BID, it is important to consider the family environment as it also has an influence on children’s and adolescents’ development of body image. Previous research has shown that a positive family environment could attenuate the associations between DM use and BID, while a negative one could amplify the association [6]. However, studies investigating the family environment as a potential moderator remain scarce.

To address the aforementioned gaps, this study investigated associations between time spent with different DM and BID in adolescents across nine European countries, exploring potential differences by sex and weight status. Furthermore, we identified latent patterns of DM use, which were then investigated in association with BID, considering the interaction effect of family environment.

## Methods

### Analysis group

This cross-sectional study included adolescents aged 12–17 years (*n* = 3854) participating in the I.Family study, the third wave of the Identification and prevention of Dietary- and lifestyle-induced health EFfects In Children and infantS (IDEFICS)/I.Family cohort conducted in nine European countries during 2013–2014 [15]. Participants were excluded if they did not complete the questionnaires by themselves (*n* = 21), did not answer any of the BID questions (*n* = 153), or missed one or more BID questions (*n* = 72). The final analysis group comprised 3608 adolescents. Parents and adolescents gave written informed consent. Ethical approval was obtained from the institutional review boards of all nine study centres, following the Declaration of Helsinki. The IDEFICS/I.Family cohort is registered in the UK’s Clinical Study Registry (ISRCTN registry) under ISRCTN62310987 (Date registered: 23/02/2018).

### Measures

#### Daily digital media duration

We assessed adolescents’ daily DM duration using a teen version of the core questionnaire, pre-tested for reliability and acceptability [16], based on methodology used in previous studies [17] (Supplementary Text S1). Adolescents reported their time spent with DM types, including TV/DVD/video (hereinafter TV), computer/game console (hereinafter PC), and internet on weekdays and weekends as: ‘not at all’, ‘< 30 min/day’, ‘30 min to 1 h/day’, ‘about 1–2 h/day’, ‘about 2–3 h/day’, and ‘>3 h/day’. Internet duration had an additional response option: ‘I’m online more or less all day/night’. To avoid overlap with internet duration, PC duration was assessed as time spent with a computer/game console per day, excluding internet time. The daily duration of each DM type, except smartphone, was calculated separately as the weighted average of the duration midpoints reported for weekdays and for weekend days, expressed in total hours/week and then converted into total hours/day to allow for continuous analysis. Daily smartphone duration was assessed by using information on the time spent with smartphones watching TV shows, movies or music videos on the previous day. The responses ranged from 0 (‘not at all’) to 5 (‘> 3 h/day’) and were then converted to duration in h/day.

#### Body image dissatisfaction

Body image dissatisfaction (BID) was assessed using four items from the Eating Disorder Diagnostic Scale, which was validated in healthy adolescent girls [18, 19]. An additional item was added to the scale to cover the whole weight spectrum. Using a six-point Likert scale ranging from 0 (‘not at all’) to 6 (‘extremely’), adolescents rated their perceived body image during the past three months using five questions: ‘…have you felt fat?’, ‘…have you had a definite fear that you might gain weight or become fat?’, ‘…has your weight influenced how you think about (judge) yourself as a person?’, ‘…has your shape influenced how you think about (judge) yourself as a person?’, and ‘…have you felt too thin?’ (Supplementary Text S2). A composite BID score was calculated across all items, ranging between 0 and 30. A higher score indicated greater BID. The internal consistency of the BID scale was satisfactory (Cronbach’s alpha = 0.81).

#### Covariates

The covariates were chosen based on prior literature to account for potential confounding in the associations between daily DM duration and BID [5, 12, 20]. Weight and height were measured in fasting status and light clothing. Height was measured to the nearest 0.1 cm using the portable stadiometer (Seca GmbH & Co. KG., Hamburg, Germany). Weight was measured to the nearest 0.1 kg using the Tanita scale (TANITA Europe GmbH, Sindelfingen, Germany). Body mass index (BMI) was calculated by dividing body weight by the squared height in metres and transformed into age- and sex-specific z-scores according to the International Obesity Task Force reference [21]. Weight status, based on BMI, was categorised as underweight, normal weight, and overweight/obese [22]. Body fat percentage was also assessed using bioelectrical impedance analysis.

Socio-demographic variables, including age, sex (male or female), and country of residence, were self-reported by adolescents and pre-tested for reliability and acceptability [16]. Pubertal status was classified as pre-pubertal/early pubertal and pubertal; the latter was determined through the development of voice (males) and menarche (females). Maturation in Tanner stages (prepubertal, peri-pubertal, and pubertal) was self-reported by participants to complement information on pubertal status across all study centres (except Italy), based on the development of pubic hair (males) or breasts (females) [23, 24]. We assessed parental education as a proxy for socio-economic status by highest educational attainment based on the International Standard Classification of Education (ISCED) [25].

Parental BID was measured using the same questions as their children. Parental BID information was not completed by both parents for every adolescent, so only one parent’s BID score was included in the model. If both parents completed the questions, information from one parent was randomly selected. Information on ‘Currently trying to lose weight (yes/no)’ was also included as a covariate to account for adolescents’ engagement in body image management.

The perceived home atmosphere was measured using eight statements, four of which were positive (e.g., warm/caring atmosphere) and four negative (e.g., strict). Adolescents rated them on a five-point Likert scale from 0 (‘not at all’) to 4 (‘very much’). Negative items were coded inversely so that all items were scored in the same direction. A score was calculated by adding all items, ranging from 0 to 32, with higher values indicating a positive home atmosphere [26].

Adolescents’ relationship with their parents was assessed using seven items, three on parents’ knowledge of their children’s whereabouts (e.g., ‘My parents know about my daily program’) and four on the encouragement of their children’s autonomy (e.g., ‘My parents listen to my opinions’). Adolescents rated statements on a 4-point Likert scale from 0 (‘rarely or never’) to 4 (‘almost always’), and a score was calculated ranging from 0 to 28. Higher scores indicate a better relationship with parents [26].

### Statistical analyses

Descriptive characteristics of participants are provided as frequencies (n) and percentages for categorical variables, and mean and standard deviation (SD) for continuous variables, stratified by sex. We performed multiple imputation using the Fully Conditional Specification (FCS) method with 10 replications to account for missing values. FCS has demonstrated unbiased handling of missing values and allows continuous and categorical variables to be included in the same model [27]. All variables were included in the multiple imputation model, except for the outcome, because the BID questions were addressed only to adolescents, meaning participants of the I.Family study aged ≥ 12 years. Therefore, > 50% of the whole I.Family study population did not have data on BID. The proportion of missing values ranged from 0% for sex and age to 22% for maturation in Tanner stages. The presented results are based on the imputed dataset of 36,080 observations (i.e., 3608 participants). Supplementary Table S1 displays the characteristics of the imputed and non-imputed samples.

To examine the association between daily DM duration and BID, linear regression models were separately performed for each DM type. All models were adjusted for sex, age, parental education attainment, country, parental BID, weight status, currently trying to lose weight, perceived home atmosphere, parental relationship, and pubertal status.

#### Latent class analyses

To identify underlying patterns of DM use, we conducted latent class analyses (LCA) based on duration categories of each DM variable (low duration: <1 h/day; medium: 1–2 h/day; high: >2 h/day). These cut-offs were set to assess trends of DM use (i.e., non-linear effects) and to enable assessing whether or not the media time recommendations of 2 h maximum per day are met [28, 29]. Adherence to this recommendation has been associated with a reduction in childhood overweight/obesity [30]. LCA was performed using two to six latent profiles of four variables (TV, PC, smartphone, and internet). Models were compared based on the Bayesian information criterion (BIC), a clear distinction of latent profiles in terms of conditional probabilities, and entropy [31, 32]. The identified latent DM profiles were then used as exposure variables in linear regression models in association with BID, adjusting for covariates. Furthermore, to examine whether a family environment moderates the association between DM profiles and BID, we added interaction terms with home atmosphere (DM profiles x home atmosphere) and parental relationship (DM profiles x parental relationship) in separate models.

#### Sex- and weight status-stratified analyses

Sex- and weight status-stratified analyses were conducted to observe potential differences in the association between daily DM duration and BID. All models were adjusted for the above-mentioned covariates.

#### Sensitivity analyses

Complete case analyses (*n* = 1,577 participants) were conducted as a sensitivity analysis to observe changes in the associations investigated. As weight status was based on BMI, which is still widely debated as being an accurate representation of body composition, body fat percentage was used instead for the main models [33].

The statistical significance level was set at α = 0.05. Unstandardised regression coefficients (β) and 95% confidence intervals (95%CI) were calculated. All statistical analyses were performed in SAS version 9.4 (Statistical Analyses System, SAS Institute Inc., Cary, NC, USA).

## Results

Overall, 3,608 adolescents were included, 51% of which were females. Mean age was similar among males (mean = 13.6, SD = 1.09) and females (mean = 13.7, SD = 1.1). Most participants had parents with a high educational attainment (males: 49.4%; females: 49.9%). In total, 29.4% of males and 25.0% of females were identified with overweight/obesity. Daily DM duration was similar between sexes for internet (males: mean = 2.0 h/day, SD = 1.5; females: mean = 2.0 h/day, SD = 1.6) and TV (males: mean = 1.8 h/day, SD = 1.0; females: mean = 1.7 h/day, SD = 1.0). However, females used their smartphones for longer (1.3 h/day, SD = 1.4) than males (1.0 h/day, SD = 1.2), while males used PC for longer (1.4 h/day, SD = 1.1) than females (0.9 h/day, SD = 0.9). The mean BID score in our analysis group was relatively low (mean = 5.8, SD = 6.4), but higher for females (mean = 7.2, SD = 6.9, range: 0–27) than males (mean = 4.3, SD = 5.4, range: 0–30) (Table 1). Since the BID score is an adapted scale, no normative cut-offs are available, however, due to the scores being near the lower possible range, it can be considered as a relatively low BID score. Supplementary Table S2 shows that adolescents in the lowest 25th percentile of BID score (≤ 1) were mostly male (61.8%) and had lower rates of overweight/obesity (13.3%) compared to the adolescents within the 75th percentile (≥ 9), who were predominantly female (68.9%) and with overweight/obesity (48.3%). Furthermore, mean duration of smartphone and internet use was higher in the 75th percentile group (smartphone: mean = 1.5, SD = 1.3; internet: mean = 2.4, SD = 1.6) than in the 25th percentile group (smartphone: mean = 1.0, SD = 1.2; internet: mean = 1.8, SD = 1.4).

**Table 1:** Characteristics of the analysis group ^1^^,^^a^

Characteristics		Males (*n* = 17640)		Females (*n* = 18440)		All (*n* = 36080)	
		N	%	N	%	N	%
Country							
Italy		3020	17.1	3020	16.4	6040	16.7
Estonia		2380	13.5	2850	15.5	5230	14.5
Cyprus		4700	26.6	4390	23.8	9090	25.2
Belgium		310	1.8	450	2.4	760	2.1
Poland		310	1.8	440	2.4	750	2.1
Sweden		1340	7.6	1390	7.5	2730	7.6
Germany		2440	13.8	2670	14.5	5110	14.2
Hungary		2410	13.7	2320	12.6	4730	13.1
Spain		730	4.1	910	4.9	1640	4.6
Parental education level							
Low		1054	6.0	1060	5.7	2114	5.9
Middle		7875	44.6	8176	44.3	16,051	44.5
High		8711	49.4	9204	49.9	17,915	49.7
Weight status							
Underweight		1197	6.8	1369	7.4	2566	7.1
Normal Weight		11,251	63.8	12,459	67.6	23,710	65.7
Overweight/Obese		5192	29.4	4612	25.0	9804	27.2
		Mean	SD	Mean	SD	Mean	SD
Age		13.6	1.1	13.7	1.1	13.6	1.1
Daily digital media duration (hours/day)							
TV		1.8	1.0	1.7	1.0	1.7	1.0
PC		1.4	1.1	0.9	0.9	1.0	1.1
Internet		2.0	1.5	2.0	1.6	2.0	1.5
Smartphone		1.0	1.2	1.3	1.4	1.1	1.3
Body image dissatisfaction score		4.3	5.4	7.2	6.9	5.8	6.4

*Abbreviations:TV* Television viewing, *PC* Computer/game console use

^1^Due to rounding, numbers might not be equal to 100%

^a^ Frequencies and mean values are calculated for the imputed dataset with 10 replications

### Associations of daily DM duration with BID

The adjusted models showed that daily smartphone and internet duration were positively associated with BID score (smartphone: β = 0.44, 95%CI: 0.31, 0.57; internet: β = 0.40, 95%CI: 0.29, 0.52) among all adolescents. This means that each additional hour spent with the smartphone was associated with a 0.44-point increase in the BID score. Daily TV and PC use were also positively associated with BID score (Table 2).

**Table 2:** Associations between daily DM duration and BID score in adolescents

DM duration (hours/day)		Crude β [95%CI]	Adjusted β^a^ [95%CI]
TV		**0.43 [0.23**,** 0.63]**	**0.19 [0.02**,** 0.35]**
PC		-0.03 [-0.23, 0.16]	**0.21 [0.04**,** 0.38]**
Internet		**0.75 [0.62**,** 0.88]**	**0.40 [0.29**,** 0.52]**
Smartphone		**0.90 [0.74**,** 1.05]**	**0.44 [0.31**,** 0.57]**

All models were conducted in the imputed dataset with 10 replications. Statistical significance based on 95%CI is shown in bold

Note: *DM* Digital media, *BID* Body image dissatisfaction, *TV* Television viewing, *PC* Computer/game console use

^a^ Linear regression models were adjusted for age, sex, weight status, country, parental education attainment, pubertal status, currently trying to lose weight, parental BID, home atmosphere, and parental relationship. β estimates are not reported for covariates

### Sex-stratified associations of daily DM duration with BID

In both males and females, daily smartphone and internet duration were positively associated with BID score (smartphone: β_males_ = 0.38, 95%CI: 0.20, 0.57; β_females_ = 0.44, 95%CI: 0.25, 0.63; internet: β_males_ = 0.28, 95%CI: 0.13, 0.43; β_females_ = 0.46, 95%CI: 0.29, 0.63, respectively). Remarkably, for females, but not for males, TV use was associated with higher BID score (TV: β_males_ = 0.07, 95%CI: -0.14, 0.28; β_females_ = 0.32, 95%CI: 0.06, 0.58) (Table 3).

**Table 3:** Associations between daily DM duration and BID score in adolescents, stratified by sex

		Adjusted Model^a^	
		Males (*n* = 17640)	Females (*n* = 18440)
DM duration (hours/day)		β [95%CI]	β [95%CI]
TV		0.07 [-0.14, 0.28]	**0.32 [0.06**,** 0.58]**
PC		**0.23 [0.03**,** 0.43]**	0.27 [-0.02, 0.57]
Internet		**0.28 [0.13**,** 0.43]**	**0.46 [0.29**,** 0.63]**
Smartphone		**0.38 [0.20**,** 0.57]**	**0.44 [0.25**,** 0.63]**

All models were conducted in the imputed dataset with 10 replications. Statistical significance based on 95%CI is shown in bold

Note: *DM* Digital media, *BID* Body image dissatisfaction, *TV* Television viewing, *PC* Computer/game console use

^a^ Linear regression models were adjusted for age, weight status, country, parental education attainment, pubertal status, currently trying to lose weight, parental BID, home atmosphere, and parental relationship. β estimates are not reported for covariates

### Weight status-stratified associations of daily DM duration with BID

Results in Table 4 showed that daily smartphone duration was positively associated with BID for all adolescents, independent of their weight status (β_underweight_ = 0.62, 95%CI: 0.14, 1.11; β_normal weight_ = 0.46, 95%CI: 0.31, 0.61; β_overweight/obese_ = 0.35, 95%CI: 0.05, 0.65), with higher estimates among adolescents with underweight and normal weight. Similarly, internet duration was positively associated with BID among all adolescents. PC duration was positively associated with BID among adolescents with underweight and normal weight (β_underweight_ = 0.52, 95%CI: 0.001, 1.03; β_normal weight_ = 0.24, 95%CI: 0.05, 0.43) but not adolescents with overweight/obesity (β_overweight/obese_ = 0.05, 95%CI: -0.34, 0.44).

**Table 4:** Associations between daily DM duration and BID score in adolescents, stratified by weight status

		Adjusted Model^a^		
		Underweight (*n* = 2566)	Normal weight (*n* = 23710)	Overweight/Obese (*n* = 9804)
DM duration (hours/day)		β [95%CI]	β [95%CI]	β [95%CI]
TV		0.43 [-0.11, 0.97]	0.17 [-0.02, 0.35]	0.19 [-0.20, 0.58]
PC		**0.52 [0.001**,** 1.03]**	**0.24 [0.05**,** 0.43]**	0.05 [-0.34, 0.44]
Internet		**0.40 [0.04**,** 0.77]**	**0.41 [0.29**,** 0.54]**	**0.32 [0.05**,** 0.60]**
Smartphone		**0.62 [0.14**,** 1.11]**	**0.46 [0.31**,** 0.61]**	**0.35 [0.05**,** 0.65]**

All models were conducted in the imputed dataset with 10 replications. Statistical significance based on 95%CI is shown in bold

Note: *DM* Digital media, *BID* Body image dissatisfaction, *TV* Television viewing, *PC* Computer/game console use

^a^ Linear regression models were adjusted for age, sex, country, parental education attainment, pubertal status, currently trying to lose weight, parental BID, home atmosphere, and parental relationship. β estimates are not reported for covariates

### Associations between latent profiles of DM duration and BID

The LCA model with four latent profiles showed the lowest BIC, clear distinction of the profiles, and higher entropy (Supplementary Table S3). The LCA models with five or six latent profiles were not chosen due to overfitting of the profiles, albeit even lower BIC values. Latent profiles were named based on the highest probabilities in each class. An overview of the conditional probabilities of all latent profiles is presented in Supplementary Table S4. About 15% of participants were included in latent profile 1, showing a low PC (< 1 h/day) and a medium TV duration (1–2 h/day) but high internet and smartphone duration (> 2 h/day). Profile 2 consisted of participants (23%) with high TV duration, internet and PC duration and low smartphone duration. Profile 3 included participants (34%) with low duration of all DM types. In profile 4, about 28% of adolescents had a low PC and smartphone duration combined with a medium TV and internet duration. For further analyses, profile 3 served as the reference. Associations between the latent DM patterns and BID are shown in Table 5. Profile 1 and profile 2 were significantly associated with a more than 1-point increase in the BID score (profile 1: β = 1.24, 95%CI: 0.73, 1.74; profile 2: β = 1.31, 95%CI: 0.83, 1.80) compared to low duration of all DM. Profile 4 showed a positive yet small association with BID.

**Table 5:** Associations between latent profiles of daily DM duration and BID score in adolescents and interaction terms

			Adjusted Model^a^
Latent profiles of DM use (Ref. Profile 3 - low duration of all media)			β [95%CI]
Profile 1: High smartphone and internet, medium TV, and low PC duration			**1.24 [0.73**,** 1.74]**
Profile 2: High TV, internet and PC, low smartphone duration			**1.31 [0.83**,** 1.80]**
Profile 4: Low smartphone and PC, medium TV and internet duration			0.26 [-0.19, 0.70]
Profile 1 x home atmosphere			**-0.11 [-0.22**,** -0.01]**
Profile 2 x home atmosphere			0.01 [-0.09, 0.10]
Profile 4 x home atmosphere			-0.06 [-0.16, 0.04]
Profile 1 x parental relationship			**-0.17 [-0.30**,** -0.03]**
Profile 2 x parental relationship			-0.004 [-0.13, 0.12]
Profile 4 x parental relationship			-0.08 [-0.20, 0.05]

All models were conducted in the imputed dataset with 10 replications. Statistical significance based on 95%CI is shown in bold

Note: *DM* Digital media, *BID* Body image dissatisfaction, *TV* Television viewing, *PC* Computer/game console use

^a^ Linear regression models were adjusted for age, sex, weight status, country, parental education attainment, pubertal status, currently trying to lose weight, parental BID, home atmosphere, and parental relationship. β estimates are not reported for covariates

### Interaction analyses

Home atmosphere interacting with profile 1 showed a negative association, suggesting an attenuating role in the association between profile 1 and BID (β_Profile1xHomeatmosphere_ = -0.11, 95%CI: -0.22, -0.01) (Table 5). The interaction between parental relationship and profile 1 also showed a negative association, suggesting a mitigating role on BID (β_Profile1xParentalrelationship_ = -0.17, 95%CI: -0.30, -0.03). No interaction effects were observed for the other profiles with any of the family environment variables.

### Sensitivity analyses

The results of the complete case analyses showed similar associations as with imputed analyses, although slightly attenuated for daily TV and PC duration, but increased for smartphone and internet duration (Supplementary Table S5). Furthermore, similar associations were observed for all DM types when the main model was adjusted for body fat percentage (Supplementary Table S6).

## Discussion

Our pan-European study showed that smartphone and internet duration were positively associated with BID among adolescents, particularly females and adolescents with underweight and normal weight status. These findings are in line with previous research, mainly conducted in females in Western countries, suggesting that time spent with online media is associated with BID among adolescents [11]. Although data collection of this study happened a decade ago, we are still able to see similarities in the findings with existing research focusing on social media (SM) use. This may be particularly explained by content promoting idealised body ideals online, which are ubiquitously accessed by adolescents via internet and smartphones.

However, research on the relations between smartphone and BID [34] or body esteem dimensions [35] is inconsistent. A previous study found that smartphone duration per se (i.e., without SM use) was neither directly nor indirectly associated with negative body esteem, even after mediation by internalisation and social comparison [36]. In contrast, our study observed a positive association between smartphone duration and BID. This discrepancy may be explained by our lack of differentiation in smartphone use and the actual content viewed online, as adolescents may have used their smartphones to engage with SM.

Our findings agree with some previous studies where prolonged TV duration was associated with BID among female adolescents [12, 37]. However, other studies have suggested that it is not the length of TV exposure but rather the content viewed that is associated with a negative body image in adolescents [38, 39]. Prolonged PC use without internet access was also positively associated with BID among European adolescents in our study. This may be partially explained by the exposure to body ideal content in video games that may promote certain body ideals [40].

Sex-specific differences revealed that longer TV, smartphone, and internet use showed stronger associations with BID in females than males, as previously reported [11, 12]. This could result from TV content emphasising certain beauty standards for female adolescents and the hyper-sexualisation of the female body [41]. Consistent with our results, a previous study observed that smartphone use was associated with BID in both females and males [34]. Nevertheless, two studies found no association between internet use and body image among boys [12, 42]. The smaller sample sizes could account for the inconsistent findings. Hence, further research should tackle these inconsistencies, especially regarding associations in males. Particularly in the SM environment, young women and men are predominantly portrayed in prevailing socio-cultural body ideals. Frequent confrontation with these gender-specific body ideals in the digital environment may lead young people to internalise this stereotype [41].

Stratification by weight status showed differences in the association between PC duration and BID, as a positive association was observed among adolescents with underweight and normal weight but not in those with overweight/obesity. The positive association between smartphone and internet duration and BID across all weight strata indicated that longer exposure to online content promoting idealised body images or certain lifestyle patterns (e.g., fit-inspiration images) may shape adolescents’ body image perceptions, independent of their weight status [43]. Previous research suggested that the strength of the relationship between BMI and body image is influenced by the degree to which unrealistic body ideals are internalised [7]. We expected adolescents with overweight/obesity to show stronger positive associations with DM due to a higher BID resulting from increased social comparison [7]. This unexpected finding may be explained by the tendency of adolescents with overweight/obesity to misperceive their bodies and underestimate their weight, while adolescents with normal and low BMI overestimate their weight [44]. In contrast, a scoping review indicated that SM use was positively associated with BID in participants with a high BMI only [45]. Hence, further research is needed to understand the role of BMI in this association.

Although the observed associations between each DM type individually and BID were overall statistically significant, the effect sizes were relatively small. However, even the smaller effect sizes may be meaningful due to the fact that these small changes in BID may accumulate over increased time spent with DM, and especially reflecting today’s reality where adolescents spend more than 2 h/day of their leisure time with DM [46, 47].

Lastly, the LCA results confirmed the findings on the association with individual DM technologies. These findings suggest that prolonged use of all DM is associated with increased BID in adolescents. Particularly, those DM that offer appearance-related content (i.e., internet and smartphones) may encourage comparison between one’s own and the idealised body shapes, increasing dissatisfaction with one’s own appearance. However, the interaction analyses showed that, particularly in adolescents with prolonged internet and smartphone use, a positive home atmosphere and parental relationship mitigate the association with BID, supporting previous results [48]. Presumably, a positive home atmosphere and parental relationships may help adolescents to reflect on the online content and the idealised body images promoted there [49]. Additionally, higher levels of self-confidence and body satisfaction among such adolescents can be protective [50]. Nonetheless, the interaction effect we observed was relatively small, suggesting that a positive family environment alone may not fully attenuate BID. Other factors may also attenuate the abovementioned associations, such as the societal responsibility to normalise body standards or the responsibility of SM developers to design safe platforms and algorithms that do not harm adolescents’ body image. It also seems reasonable to call for more political regulations, such as the newly introduced SM ban in Australia [51].

Furthermore, it is important to acknowledge that the DM landscape has changed in comparison to a decade ago (2013/2014), when data were collected in the I.Family study. At 2013/2014, adolescents’ media use comprised TV and internet-based activities, including SM, which was also getting popular among younger generations [52, 53]. Popular SM platforms were Facebook, YouTube, and the early stages of Instagram, where the feeds were not as algorithmically curated and personalised as today [54, 55]. Curated pictures and text-based posts were at the core design of SM platforms in 2013/2014. Since then, adolescents engage largely with video- and image-based SM platforms, such as TikTok or Instagram, increasing the time they spend on various platforms [56–58]. Therefore, the observed associations may be even more pronounced today and our findings offer valuable insights into the association between different DM types and BID among adolescents. Our findings are similar with research using more current data showing that SM use is associated with BID, indicating that the associations remain despite changes in the DM landscapes [58, 59].

### Strengths and limitations

This study is the first to investigate the association between time spent with different DM and BID in a large sample of European adolescents while considering underlying patterns of DM use. One key strength of this study is its large multinational sample size, including adolescents from diverse cultural backgrounds across nine European countries. Another aspect of this research is the comprehensive exploration of the relationship between time spent with four DM types and BID. By considering the influence of various media, this study provides a systemic perspective on how each DM may affect adolescents’ perceptions of body image. Including the family environment in the interaction analyses also contributed to understand its moderating role within these associations.

Nevertheless, this study has its limitations. The cross-sectional design precludes establishing causal relationships between DM and BID. Hence, reverse causation cannot be excluded. As the study relies on self-reports, the data may be biased, particularly due to social desirability and recall bias. Nonetheless, it is noteworthy that at time of data collection, methods to objectively assess DM use (e.g., data logs or screen time information from the in-built smartphone app) were not yet available. The questions used in the I.Family study were therefore appropriate for capturing adolescents’ duration of DM use. Self-reported assessment remains limited and prone to bias, but recent studies that compared self-reported and objectively measured smartphone use showed a moderate agreement for these two methods, suggesting that self-reported data on DM duration may be adequate [60]. Also, data on DM was limited to duration, which does not provide information on the content, restricting the ability to analyse content-related factors that might contribute to BID. The study by Yang et al. showed that it smartphone appis important to decipher certain smartphone activities in order to see which activities are associated with BID [36]. Further, we could not assess SM exposure. However, it is important to consider that the I.Family study was planned in 2012 and validated questionnaires on SM use were not available. Furthermore, during the early 2010s SM, such as Facebook, were accessed through the internet either via the PC or smartphone, hence internet use can be seen as the proxy of the exposure to SM [61]. Finally, the representativeness of the I.Family sample may impact the generalisability of results since most of the adolescents have parents with medium to high educational attainment. Therefore, caution should be exercised when extending the findings to broader population groups.

## Conclusion

This study based on data from 2013/2014 highlights the positive association between daily DM and BID in adolescents, particularly through smartphones and internet. Our results show that both male and female adolescents may be affected, independent of weight status. Furthermore, the findings have implications for parents and families, as they underline the important role of the parental relationship and home atmosphere in the above relationships. Promoting supportive family environments and body-positive environments, both online and offline, is essential to counteract the impact of unrealistic body standards perpetuated by DM exposure. Continued longitudinal research in this area is vital to inform (digital) interventions, creating a more body-positive environment for adolescents in the digital age.

## Supplementary Information

Supplementary Material 1.

## Data Availability

Due to the prospective nature of this cohort study, the full anonymisation of study data is ruled out and use of data requires a mutual agreement between our study consortium and interested third parties on a case-by-case basis. For corresponding requests, please contact the I.Family consortia (http://www.ifamilystudy.eu/).

## Abbreviations

BIC: Bayesian information criterion
BID: Body image dissatisfaction
BMI: Body mass index
CI: Confidence interval
DM: Digital media
FCS: Fully conditional specification
IDEFICS: Identification and prevention of Dietary–and lifestyle–induced health EFfects In Children and infantS
ISCED: International Standard Classification of Education
LCA: Latent class analyses
PC: Computer/game console
SD: Standard deviation
SM: Social media
TV: Television viewing

## References

[1] Quittkat HL, Hartmann AS, Dusing R, Buhlmann U, Vocks S. Body Dissatisfaction, importance of Appearance, and body appreciation in men and women over the lifespan. Front Psychiatry. 2019;10:864. 31920737 10.3389/fpsyt.2019.00864 PMC6928134

[2] Rodgers RF. Media and body image in children and adolescents. In: Burns T, Gottschalk F, editors. Education in the digital age: healthy and happy children. Paris: OECD Publishing; 2020. pp. 97–112.

[3] Bell BT, Dittmar H. Does media type matter? The role of identification in adolescent girls’ media consumption and the impact of different Thin-Ideal media on body image. Sex Roles. 2011;65(7–8):478–90.

[4] Kwon M. Media influences on body image & eating behaviors in adolescents. In: Evans YN, Docter AD, editors. Adolescent Nutrition - Assuring the needs of emerging adults. Switzerland: Springer; 2020. pp. 177–235.

[5] Markey CN. Invited commentary: why body image is important to adolescent development. J Youth Adolesc. 2010;39(12):1387–91. 20339908 10.1007/s10964-010-9510-0

[6] Roberts SR, Maheux AJ, Hunt RA, Ladd BA, Choukas-Bradley S. Incorporating social media and muscular ideal internalization into the tripartite influence model of body image: towards a modern Understanding of adolescent girls’ body dissatisfaction. Body Image. 2022;41:239–47. 35306356 10.1016/j.bodyim.2022.03.002

[7] Voelker DK, Reel JJ, Greenleaf C. Weight status and body image perceptions in adolescents: current perspectives. Adolesc Health Med Ther. 2015;6:149–58. 26347007 10.2147/AHMT.S68344 PMC4554432

[8] Thompson J, Heinberg L, Altabe M, Tantleff-Dunn S. Exacting beauty: Theory, assessment, and treatment of body image disturbance. 1st ed. Washington, DC: American Psychological Association; 1999.

[9] Sina E, Buck C, Veidebaum T, Siani A, Reisch L, Pohlabeln H, et al. Media use trajectories and risk of metabolic syndrome in European children and adolescents: the IDEFICS/I.Family cohort. Int J Behav Nutr Phys Act. 2021;18(1):134. 34663352 10.1186/s12966-021-01186-9 PMC8521295

[10] Borzekowski DL, Bayer AM. Body image and media use among adolescents. Adolesc Med Clin. 2005;16(2):289–313. 16111619 10.1016/j.admecli.2005.02.010

[11] Holland G, Tiggemann M. A systematic review of the impact of the use of social networking sites on body image and disordered eating outcomes. Body Image. 2016;17:100–10. 26995158 10.1016/j.bodyim.2016.02.008

[12] Hrafnkelsdottir SM, Brychta RJ, Rognvaldsdottir V, Chen KY, Johannsson E, Guethmundsdottir SL et al. Screen time and body image in Icelandic adolescents: Sex-Specific Cross-Sectional and longitudinal associations. Int J Environ Res Public Health. 2022;19(3):1308. 10.3390/ijerph19031308 PMC8835238 35162330

[13] Harriger JA, Thompson JK, Tiggemann M, TikTok. TikTok, the time is now: future directions in social media and body image. Body Image. 2023;44:222–6. 36739627 10.1016/j.bodyim.2023.01.005

[14] Mulgrew KE, Volcevski-Kostas D, Rendell PG. The effect of music video clips on adolescent boys’ body image, mood, and schema activation. J Youth Adolesc. 2014;43(1):92–103. 23443315 10.1007/s10964-013-9932-6

[15] Ahrens W, Siani A, Adan R, De Henauw S, Eiben G, Gwozdz W, et al. Cohort profile: the transition from childhood to adolescence in European children-how I.Family extends the IDEFICS cohort. Int J Epidemiol. 2017;46(5):1394–j5. 28040744 10.1093/ije/dyw317 PMC5837508

[16] Suling M, Hebestreit A, Peplies J, Bammann K, Nappo A, Eiben G, et al. Design and results of the pretest of the IDEFICS study. Int J Obes (Lond). 2011;35(Suppl 1):S30–44. 21483421 10.1038/ijo.2011.33

[17] Rideout VJ, Foehr UG, Roberts DF. Generation M2: Media in the Lives of 8- to 18-Year-Olds 2010. Available from: https://www.kff.org/other/event/generation-m2-media-in-the-lives-of/. 21 July 2025.

[18] Stice E, Telch CF, Rizvi SL. Development and validation of the eating disorder diagnostic scale: a brief self-report measure of anorexia, bulimia, and binge-eating disorder. Psychol Assess. 2000;12(2):123–31. 10887758 10.1037//1040-3590.12.2.123

[19] Stice E, Fisher M, Martinez E. Eating disorder diagnostic scale: additional evidence of reliability and validity. Psychol Assess. 2004;16(1):60–71. 15023093 10.1037/1040-3590.16.1.60

[20] Solano-Pinto N, Sevilla-Vera Y, Fernandez-Cezar R, Garrido D. Can parental body dissatisfaction predict that of children? A study on body dissatisfaction, body mass Index, and desire to diet in children aged 9–11 and their families. Front Psychol. 2021;12:650744. 33868129 10.3389/fpsyg.2021.650744 PMC8044941

[21] Cole TJ, Bellizzi MC, Flegal KM, Dietz WH. Establishing a standard definition for child overweight and obesity worldwide: international survey. BMJ. 2000;320(7244):1240. 10797032 10.1136/bmj.320.7244.1240 PMC27365

[22] Cole TJ, Lobstein T. Extended international (IOTF) body mass index cut-offs for thinness, overweight and obesity. Pediatr Obes. 2012;7(4):284–94. 22715120 10.1111/j.2047-6310.2012.00064.x

[23] Marshall WA, Tanner JM. Variations in pattern of pubertal changes in girls. Arch Dis Childh. 1969;44:291–303. 5785179 10.1136/adc.44.235.291 PMC2020314

[24] Marshall WA, Tanner JM. Variations in the pattern of pubertal changes in boys. Arch Dis Childh. 1970;45:13–23. 5440182 10.1136/adc.45.239.13 PMC2020414

[25] United Nations Educational Scientific and Cultural Organization. International standard classification of education (ISCED) 2011. Montreal; 2012. Available from: https://unesdoc.unesco.org/ark:/48223/pf0000219109/PDF/219109eng.pdf.multi. 24 May 2025.

[26] Latendresse SJ, Rose RJ, Viken RJ, Pulkkinen L, Kaprio J, Dick DM. Parental socialization and adolescents’ alcohol use behaviors: predictive disparities in parents’ versus adolescents’ perceptions of the parenting environment. J Clin Child Adolesc Psychol. 2009;38(2):232–44. 19283601 10.1080/15374410802698404 PMC3641770

[27] Liu Y, De A. Multiple imputation by fully conditional specification for dealing with missing data in a large epidemiologic study. Int J Stat Med Res. 2015;4(3):287–95. 27429686 10.6000/1929-6029.2015.04.03.7 PMC4945131

[28] Bundesinsitut für öffentliche Gesundheit. Medien und Digitales - Elterninfo 2022. Available from: https://shop.bioeg.de/pdf/11041410.pdf. 27 June 2025.

[29] Canadian Paediatric Society. How much for adolescents? (12–17) 2024. Available from: https://cps.ca/en/active-actifs/how-much-for-adolescents. 27 June 2025.

[30] Bornhorst C, Pigeot I, De Henauw S, Formisano A, Lissner L, Molnar D, et al. The effects of hypothetical behavioral interventions on the 13-year incidence of overweight/obesity in children and adolescents. Int J Behav Nutr Phys Act. 2023;20(1):100. 37620898 10.1186/s12966-023-01501-6 PMC10463721

[31] Lanza ST, Collins LM, Lemmon DR, Schafer JL. PROC LCA: A SAS procedure for latent class analysis. Struct Equ Model. 2007;14(4):671–94. 10.1080/10705510701575602 PMC2785099 19953201

[32] Weller BE, Bowen NK, Faubert SJ. Latent class analysis: A guide to best practice. J Black Psychol. 2020;46(4):287–311.

[33] Nuttall FQ. Body mass index: Obesity, BMI, and health: A critical review. Nutr Today. 2015;50(3):117–28. 27340299 10.1097/NT.0000000000000092 PMC4890841

[34] Kwon S, Kim R, Lee JT, Kim J, Song S, Kim S, et al. Association of smartphone use with body image distortion and weight loss behaviors in Korean adolescents. JAMA Netw Open. 2022;5(5):e2213237. 35594044 10.1001/jamanetworkopen.2022.13237 PMC9123497

[35] Lo Coco G, Salerno L, Giordano C, Di Blasi M, Rodgers RF. Understanding the smartphone generation: is problematic smartphone use associated with low body esteem among adolescent girls and boys? Curr Psychol. 2020;41(5):3173–84.

[36] Yang H, Wang JJ, Tng GYQ, Yang S. Effects of social media and smartphone use on body esteem in female adolescents: testing a cognitive and affective model. Child (Basel). 2020;7(9):148. 10.3390/children7090148 PMC7552652 32967376

[37] Schooler D, Trinh S. Longitudinal associations between television viewing patterns and adolescent body satisfaction. Body Image. 2011;8(1):34–42. 21050831 10.1016/j.bodyim.2010.09.001

[38] López-Guimerà G, Levine MP, Sánchez-Carracedo D, Fauquet J. Influence of mass media on body image and eating disordered attitudes and behaviors in females: A review of effects and processes. Media Psychol. 2010;13(4):387–416.

[39] Tiggemann M. Television and adolescent body image: the role of program content and viewing motivation. J Soc Clin Psychol. 2005;24(3):361–81.

[40] Barlett CP, Harris RJ. The impact of body emphasizing video games on body image concerns in men and women. Sex Roles. 2008;59(7–8):586–601.

[41] Santoniccolo F, Trombetta T, Paradiso MN, Rolle L. Gender and media representations: A review of the literature on gender Stereotypes, objectification and sexualization. Int J Environ Res Public Health. 2023;20(10):5770. 10.3390/ijerph20105770 PMC10218532 37239498

[42] Anez E, Fornieles-Deu A, Fauquet-Ars J, Lopez-Guimera G, Punti-Vidal J, Sanchez-Carracedo D. Body image dissatisfaction, physical activity and screen-time in Spanish adolescents. J Health Psychol. 2016;23(1):36–47. 27557652 10.1177/1359105316664134

[43] Tiggemann M, Zaccardo M. Strong is the new skinny’: A content analysis of #fitspiration images on Instagram. J Health Psychol. 2018;23(8):1003–11. 27611630 10.1177/1359105316639436

[44] Plotas P, Tsekoura E, Souris E, Kantanis A, Kostopoulou E, Varvarigou A et al. Body-Size Misperception among Overweight Children and Adolescents in Greece: A Cross-Sectional Study. Nutrients. 2023;15(8):1814. 10.3390/nu15081814 PMC10145176 37111033

[45] Dane A, Bhatia K. The social media diet: A scoping review to investigate the association between social media, body image and eating disorders amongst young people. PLOS Glob Public Health. 2023;3(3):e0001091. 36962983 10.1371/journal.pgph.0001091 PMC10032524

[46] Orgiles M, Amoros-Reche V, Francisco R, Godinho C, Delvecchio E, Mazzeschi C, et al. Beyond the pandemic: tracing the evolution of activity, screen time, and sleep in European children over 3 years. Eur J Pediatr. 2025;184(10):629. 40986123 10.1007/s00431-025-06458-1 PMC12457492

[47] Wanka F, Vogel M, Grafe N, Assmann M, Kiess W, Poulain T. Leisure activities of adolescents-associations with demographic characteristics, well-being and parental leisure engagement. Pediatr Res. 2025;98(2):559–67. 39843778 10.1038/s41390-025-03866-9 PMC12454157

[48] de Vries DA, van der Vossen HGM. Kolk-van der boom P. Social media and body dissatisfaction: investigating the attenuating role of positive Parent-Adolescent relationships. J Youth Adolesc. 2019;48(3):527–36. 30478819 10.1007/s10964-018-0956-9 PMC6394528

[49] Tylka TL. Positive psychology perspectives on body image. In: Cash TF, editor. Encyclopedia of body image and human appearance. 1st ed. Boston: Elsevier Academic Press; 2012. pp. 657–63.

[50] O’Dea JA. Body image and self-esteem. In: Cash TF, editor. Encyclopedia of body image and human appearance. 1st ed. Boston: Elsevier Academic Press; 2012. pp. 141–7.

[51] Fardouly J. Potential effects of the social media age ban in Australia for children younger than 16 years. Lancet Digit Health. 2025;7(4):e235–6. 40148007 10.1016/j.landig.2025.01.016

[52] Twenge JM, Martin GN, Spitzberg BH. Trends in U.S. Adolescents’ media use, 1976–2016: the rise of digital media, the decline of TV, and the (near) demise of print. Psychol Popular Media Cult. 2019;8(4):329–45.

[53] Tsitsika AK, Tzavela EC, Janikian M, Olafsson K, Iordache A, Schoenmakers TM, et al. Online social networking in adolescence: patterns of use in six European countries and links with psychosocial functioning. J Adolesc Health. 2014;55(1):141–7. 24618179 10.1016/j.jadohealth.2013.11.010

[54] Vandenbosch L, Fardouly J, Tiggemann M. Social media and body image: recent trends and future directions. Curr Opin Psychol. 2022;45:101289. 35030460 10.1016/j.copsyc.2021.12.002

[55] Dekker CA, Baumgartner SE, Sumter SR. For you vs. for everyone: the effectiveness of algorithmic personalization in driving social media engagement. Telematics Inform. 2025;101:102300.

[56] Qi J, Yan Y, Yin H. Screen time among school-aged children of aged 6–14: a systematic review. Glob Health Res Policy. 2023;8(1):12. 37076910 10.1186/s41256-023-00297-z PMC10113131

[57] Choi EJ, King GKC, Duerden EG. Screen time in children and youth during the pandemic: A systematic review and meta-analysis. Global Pediatr. 2023;6:100080.

[58] Saiphoo AN, Vahedi Z. A meta-analytic review of the relationship between social media use and body image disturbance. Comput Hum Behav. 2019;101:259–75.

[59] Sagrera CE, Magner J, Temple J, Lawrence R, Magner TJ, Avila-Quintero VJ, et al. Social media use and body image issues among adolescents in a vulnerable Louisiana community. Front Psychiatry. 2022;13:1001336. 36405904 10.3389/fpsyt.2022.1001336 PMC9669337

[60] Coyne P, Voth J, Woodruff SJ. A comparison of self-report and objective measurements of smartphone and social media usage. Telematics Inf Rep. 2023;10:100061.

[61] Mascheroni G, Ólafsson K. Net Children Go Mobile - Risk and opportunities: Full Findings Report. Milano; 2014. Available from: https://researchonline.lse.ac.uk/id/eprint/55798/1/Net_Children_Go_Mobile_Risks_and_Opportunities_Full_Findings_Report.pdf. 16 Dec 2025.

