# Supplementary material for: Associations between time spent with digital media and body image among European adolescents
Source: BMC Pediatr. 2026 Jan 31;26:149. doi: 10.1186/s12887-026-06551-w (PMC12930871; doi:10.1186/s12887-026-06551-w)
Supplement: Supplementary file 1 — Supplementary Material 1. [file 12887_2026_6551_MOESM1_ESM.docx]

**Supplementary material**

Associations between time spent with digital media and body image among European adolescents

| **Text S1** | Questionnaire for time spent with digital media (daily digital media duration) |
| --- | --- |
| **Text S2** | Questionnaire for body image dissatisfaction score |
| **Table S1** | Characteristics of the analysis group in the non-imputed and imputed sample |
| **Table S2** | Characteristics of the analysis group based on the 25th and 75th percentiles of the BID score |
| **Table S3** | Latent class analysis (LCA) Model Fit statistics |
| **Table S4** | Class membership probabilities and latent class profiles |
| **Table S5** | Association between daily DM duration and BID score in adolescents without missing information on the included variables (complete case analyses) |
| **Table S6** | Association between daily DM duration and BID score in adolescents, further adjusting for body fat percentage |

**Text S1|** Questionnaire for time spent with digital media (daily digital media duration) (1)

**How long do you usually watch TV and/or video/DVD per day?**

*Please tick one answer per line.*

Not at Less than 30 min.- About About More than

all 30 min. 1 hour 1-2 hrs. 2-3 hrs. 3 hours

per day per day per day per day per day

Weekdays  **⚫** _0_  **⚫** _1_  **⚫** _2_  **⚫** _3_  **⚫** _4_  **⚫** _5_

Weekends  **⚫** _0_  **⚫** _1_  **⚫** _2_  **⚫** _3_  **⚫** _4_  **⚫** _5_

**How long do you usually use the internet per day?**

*Please tick one answer per line.*

Not at Less than 30 min.- About About More than I’m online

all 30 min. 1 hour 1-2 hrs. 2-3 hrs. 3 hours more or

per day per day per day per day per day less all

day/night

Weekdays  **⚫** _0_  **⚫** _1_  **⚫** _2_  **⚫** _3_  **⚫** _4_  **⚫** _5_  **⚫** _6_

Weekends  **⚫** _0_  **⚫** _1_  **⚫** _2_  **⚫** _3_  **⚫** _4_  **⚫** _5_  **⚫** _6_

**How long do you usually sit at a computer/game console per day? (Please disregard the time spent with internet-use.)**

*Please tick one answer per line.*

Not at Less than 30 min.- About About More than

all 30 min. 1 hour 1-2 hrs. 2-3 hrs. 3 hours

per day per day per day per day per day

Weekdays  **⚫** _0_  **⚫** _1_  **⚫** _2_  **⚫** _3_  **⚫** _4_  **⚫** _5_

Weekends  **⚫** _0_  **⚫** _1_  **⚫** _2_  **⚫** _3_  **⚫** _4_  **⚫** _5_

**Thinking only about yesterday, about how much time did you spend**

***watching TV* shows, movies or music videos on any of the following? (Please disregard the time spent with computer games.)**

*Please tick one answer per line.*

Not Less than 30 min. About About More

at all 30 min. - 1 hour 1-2 hours 2-3 hours than

3 hours

Cell phone  **⚫** _0_  **⚫** _1_  **⚫** _2_  **⚫** _3_  **⚫** _4_  **⚫** _5_

**Text S2|** Questionnaire for body image dissatisfaction score (2)

**Below you find five statements concerning how you feel about your body.**

Please indicate how much each statement applied to you over the past three months from 0 (not at all) to 6 (extremely).

*Please tick one answer per line.*

Not at all Slightly Moderately Extremely

**Over the past 3 months…** **⓪ ① ② ③ ④ ⑤ ⑥**

… have you felt fat  **⚫** _0_ **⚫** _1_  **⚫** _2_  **⚫** _3_  **⚫** _4_  **⚫** _5_  **⚫** _6_

… have you had a definite fear

that you might gain weight or

become fat

… has your weight influenced

how you think about (judge)

yourself as a person

… has your shape influenced

how you think about (judge)

yourself as a person

**⚫** _0_ **⚫** _1_  **⚫** _2_  **⚫** _3_  **⚫** _4_  **⚫** _5_  **⚫** _6_

**⚫** _0_ **⚫** _1_  **⚫** _2_  **⚫** _3_  **⚫** _4_  **⚫** _5_  **⚫** _6_

**⚫** _0_ **⚫** _1_  **⚫** _2_  **⚫** _3_  **⚫** _4_  **⚫** _5_  **⚫** _6_

… have you felt too thin  **⚫** _0_ **⚫** _1_  **⚫** _2_  **⚫** _3_  **⚫** _4_  **⚫** _5_  **⚫** _6_

| Table S1 | | Characteristics of the analysis group in the non-imputed and imputed sample ^1^ | | | | | | | | | | | | | | | |
| --- | --- | --- | --- | --- | --- | --- | --- | --- | --- | --- | --- | --- | --- | --- | --- | --- | --- |
| Characteristics | | |  | | Non-imputed sample | | | | | | Complete case sample | | | | Imputed sample ^a^ | | |
|  | | |  | | N | | % | | | | N | % | | | N | % | |
| All | | | | | 3608 | | 100.0 | | | | 1577 | 100.0 | | | 36080 | 100.0 | |
| Sex | | | | |  | |  | | | |  |  | | |  |  | |
| Male | | | | | 1764 | | 48.9 | | | | 742 | 47.1 | | | 17640 | 48.8 | |
| Female | | | | | 1844 | | 51.1 | | | | 835 | 53.0 | | | 18440 | 51.2 | |
| Country | | | | | | | | | | | | | | |  |  | |
|  | Italy | | | | 604 | | 16.7 | | | | - | - | | | 6040 | 16.7 | |
|  | Estonia | | | | 523 | | 14.5 | | | | 270 | 17.1 | | | 5230 | 14.5 | |
|  | Cyprus | | | | 909 | | 25.2 | | | | 442 | 28.0 | | | 9090 | 25.2 | |
|  | Belgium | | | | 76 | | 2.1 | | | | 31 | 2.0 | | | 760 | 2.1 | |
|  | Poland | | | | 75 | | 2.1 | | | | - | - | | | 750 | 2.1 | |
|  | Sweden | | | | 273 | | 7.6 | | | | 177 | 11.2 | | | 2730 | 7.6 | |
|  | Germany | | | | 511 | | 14.2 | | | | 269 | 17.1 | | | 5110 | 14.2 | |
|  | Hungary | | | | 473 | | 13.1 | | | | 325 | 20.6 | | | 4730 | 13.1 | |
|  | Spain | | | | 164 | | 4.6 | | | | 63 | 4.0 | | | 1640 | 4.6 | |
| Parental education level | | | | | | | | | | | | | | |  |  | |
|  | Low | | | | 199 | | 5.5 | | | | 34 | 2.2 | | | 2114 | 5.9 | |
|  | Middle | | | | 1514 | | 42.0 | | | | 606 | 67.8 | | | 16051 | 44.5 | |
|  | High | | | | 1694 | | 47.0 | | | | 937 | 23.4 | | | 17915 | 49.7 | |
|  | Missing | | | | 201 | | 5.6 | | | |  |  | | |  |  | |
| Weight status | | |  |  | |  | | |  | |  |  | | |  |  |  |
|  | Underweight | | | | 271 | | 7.5 | | | | 139 | 8.8 | | | 2566 | 7.1 | |
|  | Normal weight | | | | 2360 | | 65.4 | | | | 1069 | 67.8 | | | 23710 | 65.7 | |
|  | Overweight/Obese | | | | 977 | | 27.1 | | | | 369 | 23.4 | | | 9804 | 27.2 | |
|  |  | | | | Mean | | SD | | | | Mean | SD | | | Mean | SD | |
| Age | | | | | 13.6 | | 1.1 | | | | 13.7 | 1.1 | | | 13.6 | 1.1 | |
| Daily digital media duration (hours per day) | | | | | | | | | | | | | | |  |  | |
|  | TV | | | | 1.7 | | 1.0 | | | | 1.8 | 1.0 | | | 1.7 | 1.0 | |
|  | PC | | | | 1.0 | | 1.1 | | | | 1.0 | 1.1 | | | 1.0 | 1.1 | |
|  | Internet | | | | 2.0 | | 1.6 | | | | 2.1 | 1.6 | | | 2.0 | 1.5 | |
|  | Smartphone | | | | 1.1 | | 1.3 | | | | 1.1 | 1.3 | | | 1.1 | 1.3 | |
| Body Image Dissatisfaction score | | | | | 5.8 | | | 6.4 | | 5.9 | | | 6.4 | 5.8 | | | 6.4 |
| ^1^ Due to rounding, numbers might not be equal to 100%.  ^a^ Frequencies and mean values are calculated for the imputed dataset with 10 replications  Abbreviations: TV= television viewing, PC= computer/game console use | | | | | | | | | | | | | | | | | |

| Table S2 | | Characteristics of the analysis group based on the 25^th^ and 75^th^ percentiles of the BID score ^1, a^ | | | | | | | | |
| --- | --- | --- | --- | --- | --- | --- | --- | --- | --- | --- |
| Characteristics | | |  | | 25^th^ percentile of BID score (≤1) | | | | 75^th^ percentile of BID score (≥9) | |
|  | | |  | | N | | % | | N | % |
| All | | | | | 12330 | | 100.0 | | 9550 | 100.0 |
| Sex | | | | |  | |  | |  |  |
| Male | | | | | 7620 | | 61.8 | | 2970 | 31.1 |
| Female | | | | | 4710 | | 38.20 | | 6580 | 68.9 |
| Country | | | | | | | | | | |
|  | Italy | | | | 2000 | | 16.2 | | 1700 | 17.8 |
|  | Estonia | | | | 1230 | | 10.0 | | 1950 | 20.4 |
|  | Cyprus | | | | 2790 | | 22.6 | | 2530 | 26.5 |
|  | Belgium | | | | 320 | | 2.6 | | 130 | 1.4 |
|  | Poland | | | | 400 | | 3.2 | | 50 | 0.5 |
|  | Sweden | | | | 980 | | 8.0 | | 570 | 6.0 |
|  | Germany | | | | 1760 | | 14.3 | | 1430 | 15.0 |
|  | Hungary | | | | 2100 | | 17.1 | | 940 | 9.8 |
|  | Spain | | | | 740 | | 6.0 | | 250 | 2.6 |
| Parental education level | | | | | | | | | | |
|  | Low | | | | 511 | | 4.1 | | 657 | 6.9 |
|  | Middle | | | | 5569 | | 45.2 | | 4492 | 47.0 |
|  | High | | | | 6250 | | 50.7 | | 4401 | 46.1 |
| Weight status | | |  |  | |  | |  |  |  |
|  | Underweight | | | | 1019 | | 8.3 | | 363 | 3.8 |
|  | Normal weight | | | | 9671 | | 78.4 | | 4571 | 47.9 |
|  | Overweight/Obese | | | | 1640 | | 13.3 | | 4616 | 48.3 |
|  |  | | | | Mean | | SD | | Mean | SD |
| Age | | | | | 13.5 | | 1.0 | | 13.8 | 1.1 |
| Daily digital media duration (hours per day) | | | | | | | | | | |
| TV | | | | | 1.7 | | 1.0 | | 1.9 | 1.0 |
| PC | | | | | 1.1 | | 1.1 | | 1.0 | 1.1 |
| Internet | | | | | 1.8 | | 1.4 | | 2.4 | 1.6 |
| Smartphone | | | | | 1.0 | | 1.2 | | 1.5 | 1.3 |
| ^1^ Due to rounding, numbers might not be equal to 100%.  ^a^ Frequencies and mean values are calculated for the imputed dataset with 10 replications  Abbreviations: TV= television viewing, PC= computer/game console use | | | | | | | | | | |

| **Table S3** | Latent class analysis (LCA) Model Fit statistics | | | | | | |
| --- | --- | --- | --- | --- | --- | --- | --- |
| **Nr. of classes** | | **Akaike Information Criterion (AIC)** | **Bayesian Information Criterion**  **(BIC)** | **Adjusted Bayesian Information Criterion (aBIC)** | | **Entropy** | **Model converged** |
| 2 | | 4435.28 | 4579.67 | 4525.64 | 0.64 | | Yes |
| 3 | | 1755.72 | 1976.55 | 1893.92 | 0.56 | | Yes |
| **4** | | **930.84** | **1228.11** | **1116.88** | **0.61** | | **Yes** |
| 5 | | 602.31 | 976.02 | 836.19 | 0.58 | | Yes |
| 6 | | 411.55 | 861.70 | 693.27 | 0.57 | | Yes |
|  | | | | | | | |

| **Table S4** | Class membership probabilities and latent class profiles | | | | |
| --- | --- | --- | --- | --- | --- |
| **Class** | | **Profile 1** | **Profile 2** | **Profile 3** | **Profile 4** |
| Latent Profiles | | High smartphone and internet, medium TV, and low PC duration | High TV, internet and PC, low smartphone duration | Low duration of all media | Low smartphone and PC, medium TV and internet duration |
| Class membership probabilities: Gamma estimates (standard errors) | | 0.1551 (0.0148) | 0.2268 (0.0335) | 0.3381 (0.0505) | 0.2800 (0.0783) |
| Item response probabilities: Rho estimates (standard errors) | | | | | |
| Low duration (<1 hour) | | | | | |
| TV | | 0.2123 (0.0237) | 0.1515 (0.0256) | 0.5657 (0.0460) | 0.2020 (0.0586) |
| PC | | 0.7240 (0.0519) | 0.2373 (0.0447) | 0.9428 (0.0295) | 0.5462 (0.0676) |
| Internet | | 0.1292 (0.0492) | 0.0532 (0.0184) | 0.7834 (0.0989) | 0.2423 (0.0908) |
| Smartphone | | 0.0087 (0.0157) | 0.5051 (0.0375) | 0.8970 (0.0453) | 0.8181 (0.0485) |
| Medium duration (1-2 hours) | | | | | |
| TV | | 0.4472 (0.0231) | 0.1455 (0.0205) | 0.3385 (0.0378) | 0.5256 (0.0511) |
| PC | | 0.2750(0.0464) | 0.1602 (0.0275) | 0.0420 (0.0234) | 0.3922 (0.0542) |
| Internet | | 0.2997 (0.0362) | 0.0480 (0.0132) | 0.1323 (0.0671) | 0.5143 (0.0674) |
| Smartphone | | 0.2402 (0.0982) | 0.1068 (0.0118) | 0.0606 (0.0201) | 0.1811 (0.0480) |
| High duration (>2 hours) | | | | | |
| TV | | 0.3405 (0.0353) | 0.7031 (0.0416) | 0.0958 (0.0262) | 0.2725 (0.0391) |
| PC | | 0.0010 (0.0074) | 0.6025 (0.0420) | 0.0153 (0.0090) | 0.0616 (0.0213) |
| Internet | | 0.5711 (0.0564) | 0.8988 (0.0278) | 0.0842 (0.0353) | 0.2434 (0.0448) |
| Smartphone | | 0.7511 (0.1090) | 0.3881 (0.0368) | 0.0423 (0.0324) | 0.0008 (0.0016) |

| **Table S5** | Association between daily DM duration and BID score in adolescents without missing information on the included variables (complete case analyses) | |
| --- | --- | --- |
|  | | **Adjusted Model** ^a^ |
| DM duration (hours/day) | | **β [95%CI]** |
| TV | | 0.09 [-0.16, 0.35] |
| PC | | 0.07 [-0.18, 0.31] |
| Internet | | **0.45 [0.28, 0.61]** |
| Smartphone | | **0.52 [0.31, 0.72]** |
| ^a^ Linear regression models were adjusted for age, sex, weight status, country, parental education attainment, pubertal status, currently trying to lose weight, parental BID, home atmosphere, and parental relationship. β estimates are not reported for covariates. All models were conducted in the imputed dataset with 10 replications. Statistical significance based on 95%CI is shown in bold.  Note: DM = Digital media, BID = Body image dissatisfaction, TV= television viewing, PC= computer/game console use | | |

| **Table S6** | Association between daily DM duration and BID score in adolescents, further adjusting for body fat percentage | |
| --- | --- | --- |
|  | | **Adjusted Model** ^a^ |
| DM duration (hours/day) | | **β [95%CI]]** |
| TV | | 0.15 [-0.01, 0.32] |
| PC | | **0.23 [0.06, 0.40]** |
| Internet | | **0.40 [0.28, 0.51]** |
| Smartphone | | **0.44 [0.31, 0.58]** |
| ^a^ Linear regression models were adjusted for age, sex, country, parental education attainment, pubertal status, currently trying to lose weight, parental BID, home atmosphere, and parental relationship. β estimates are not reported for covariates. All models were conducted in the imputed dataset with 10 replications. Statistical significance based on 95%CI is shown in bold.  Note: DM = Digital media, BID = Body image dissatisfaction, TV= television viewing, PC= computer/game console use | | |

***References***

1. Suling M, Hebestreit A, Peplies J, Bammann K, Nappo A, Eiben G, et al. Design and results of the pretest of the IDEFICS study. Int J Obes (Lond). 2011;35 Suppl 1:S30-44.

2. Stice E, Telch CF, Rizvi SL. Development and validation of the Eating Disorder Diagnostic Scale: a brief self-report measure of anorexia, bulimia, and binge-eating disorder. Psychol Assess. 2000;12(2):123-31.
